# Post-scabies Dermatitis Mimicking Treatment Failure: A Diagnostic Challenge

**DOI:** 10.7759/cureus.109428

**Published:** 2026-05-22

**Authors:** Sola Maria Saleh, Ramez Azzam

**Affiliations:** 1 Health Outcomes and Biomedical Informatics, College of Medicine, University of Florida, Gainesville, USA; 2 Infectious Diseases, College of Medicine, University of Florida, Gainesville, USA

**Keywords:** hypersensitivity reaction, ivermectin, permethrin, post-scabies dermatitis, pruritus, scabies

## Abstract

Scabies is a common parasitic infestation caused by *Sarcoptes scabiei* var. hominis and is typically treated effectively with topical permethrin or oral ivermectin. After successful eradication, persistent pruritus and inflammatory skin lesions may still be present, leading to diagnostic uncertainty and unnecessary retreatment. We report a case of a 69-year-old male who developed persistent pruritic erythematous papules and eczematous plaques following appropriate permethrin and ivermectin therapy for confirmed scabies. Clinical reassessment and dermatology evaluation revealed no evidence of active infestation. The patient was diagnosed with post-scabies dermatitis, a hypersensitivity reaction to retained mite antigens. Treatment with topical corticosteroids, oral antihistamines, and emollients resulted in gradual symptom resolution over several weeks. This case highlights the importance of distinguishing post-scabies dermatitis from treatment failure or reinfestation to prevent unnecessary scabicide exposure and reduce patient anxiety regarding his unresolved condition.

## Introduction

Scabies is a contagious parasitic infestation caused by *Sarcoptes scabiei* var. hominis and is characterized by intense nocturnal pruritus and erythematous papules, burrows, and excoriations in characteristic anatomical locations [1]. It is estimated that approximately 200 million people worldwide are infected by it at any given time, making it a significant public health concern, particularly in resource-limited settings [2]. Prolonged skin-to-skin contact is the main source of transmission, often affecting multiple household members [1,3].

The treatment of choice for scabies involves topical application of 5% permethrin cream or oral administration of ivermectin; both demonstrate high efficacy when used appropriately [4]. In some cases, itching may persist for weeks after successful treatment despite eradication of the mites, a phenomenon supported by recent clinical updates on scabies management [5].

The term "post-scabies dermatitis" has been used to describe the persistent inflammatory response that occurs after successful elimination of the mite [6]. This condition represents a delayed type IV hypersensitivity response to retained mite antigens, eggs, and fecal material within the skin [7]. Clinically, it may present with persistent pruritus, eczematous plaques, or nodular lesions, often raising concern for treatment failure or reinfestation [6]. Post-scabies dermatitis should be suspected in patients with persistent pruritus and inflammatory lesions following appropriate treatment, particularly when adherence is confirmed, close contacts have been treated, and no new burrows are identified. Dermoscopy can be a valuable adjunct in this setting, as the absence of the characteristic delta wing jet sign (representing the mite) supports the absence of active infestation and favors a post-inflammatory hypersensitivity reaction [8,9].

It is crucial to distinguish post-scabies dermatitis from active infestation in order to prevent unnecessary retreatment, reduce any adverse effects related to the medication, and alleviate patient anxiety regarding their unresolved condition. We present a case highlighting this diagnostic challenge and its appropriate management.

The practical value of this case lies not in the novelty of post-scabies dermatitis itself, but in emphasizing the importance of recognizing this post-treatment inflammatory reaction to avoid unnecessary repeated scabicidal therapy, treatment-related irritation, patient anxiety, and misclassification as persistent infestation, therapy-induced irritant dermatitis, nonspecific eczematous dermatitis, or secondary bacterial infection.

## Case presentation

**Figure 1:**
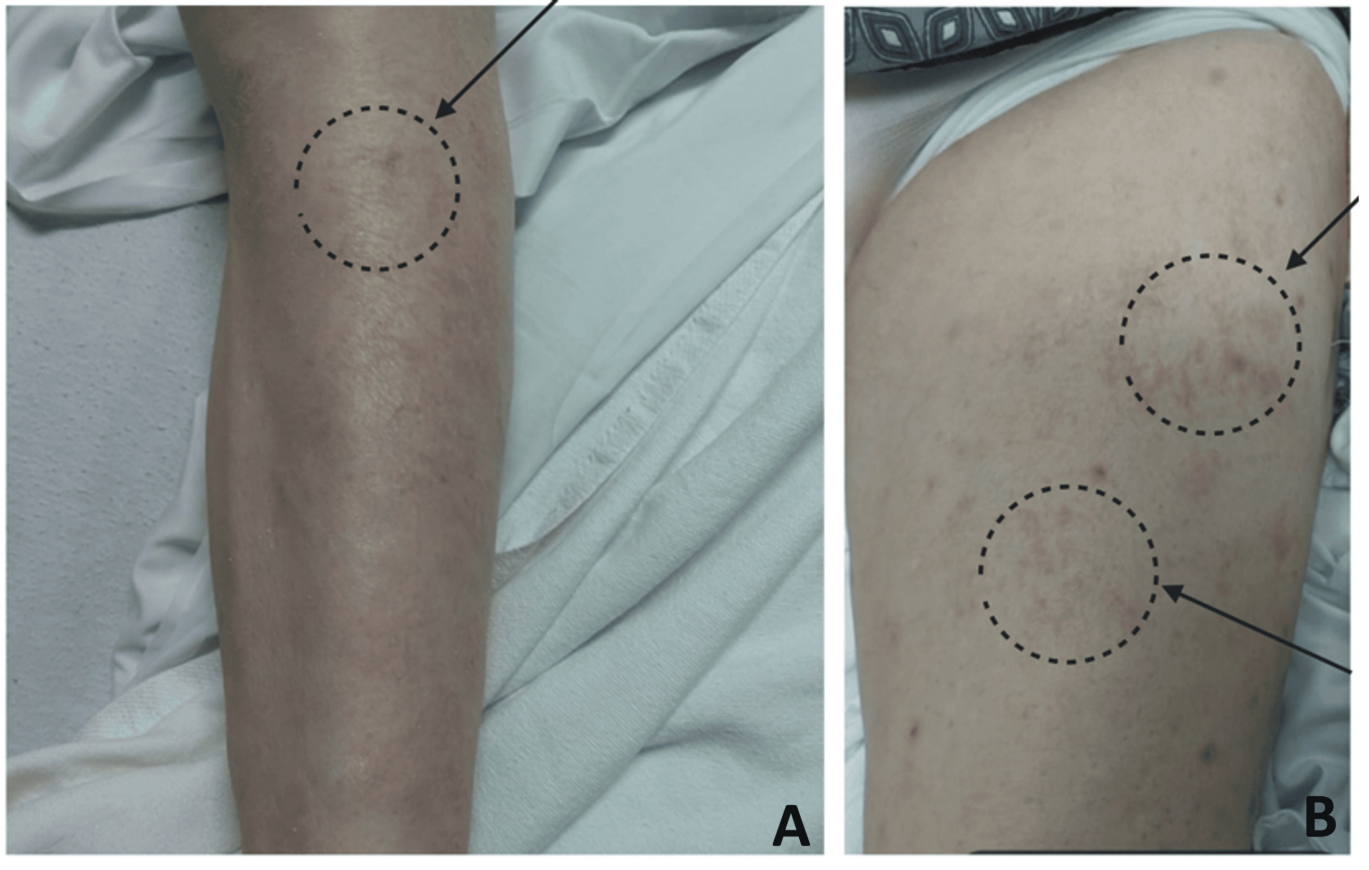
Pre-treatment clinical presentation of post-scabies dermatitis showing scattered erythematous macules and papules on the lower extremity (arrows and halos).

A 69-year-old male with past medical history of HIV on dolutegravir/lamivudine dual therapy with undetectable viral load and a CD4 count of 482 cells/µL, atrial fibrillation, chronic obstructive pulmonary disease (COPD), and type 2 diabetes mellitus (DM) presented with a two-month history of generalized pruritus, associated with erythematous papules all over his body. Several close family members had similar symptoms. All family members were diagnosed with scabies and received appropriate treatment, with complete resolution of symptoms in all except the patient. Despite completing two courses of permethrin 5% cream applied from the neck down and repeated after seven days, along with oral ivermectin administered at a dose of 200 µg/kg in two doses separated by seven days, the patient continued to experience persistent pruritus and eczematous skin changes. Clinical examination revealed diffuse, ill-defined erythematous papules, patches, and plaques involving the trunk, extremities, and face (Figures 1A, 1B). No burrows, vesicles, or new classic scabetic lesions were identified.

Dermoscopic evaluation of multiple representative lesions did not demonstrate mites, eggs, or fecal pellets (scybala), and no delta wing sign was visualized. These findings, along with appropriate prior treatment without clinical improvement, support a hypersensitivity reaction rather than active infestation. Given the absence of active infestation and the inflammatory morphology of lesions, a diagnosis of post-scabies dermatitis was made based on clinical features, treatment response, and exclusion of active infestation.

At the time of laboratory evaluation, the patient was also diagnosed with pneumonia, which caused markedly elevated inflammatory markers, including CRP and ESR. There were no clinical signs of secondary bacterial infection of the skin, including purulence, warmth, fluctuance, honey-colored crusting, rapidly progressive erythema, or systemic symptoms attributable to a cutaneous source. The laboratory abnormalities were therefore not interpreted as evidence supporting or contradicting post-scabies dermatitis, but rather as findings related to a concurrent systemic infection.

Skin scraping was not performed. Therefore, exclusion of persistent active infestation relied on the overall clinical course, completion of appropriate scabicidal therapy, treatment and clinical resolution among close contacts, absence of new burrows or new classic scabetic lesions, and negative dermoscopic findings.

**Figure 2:**
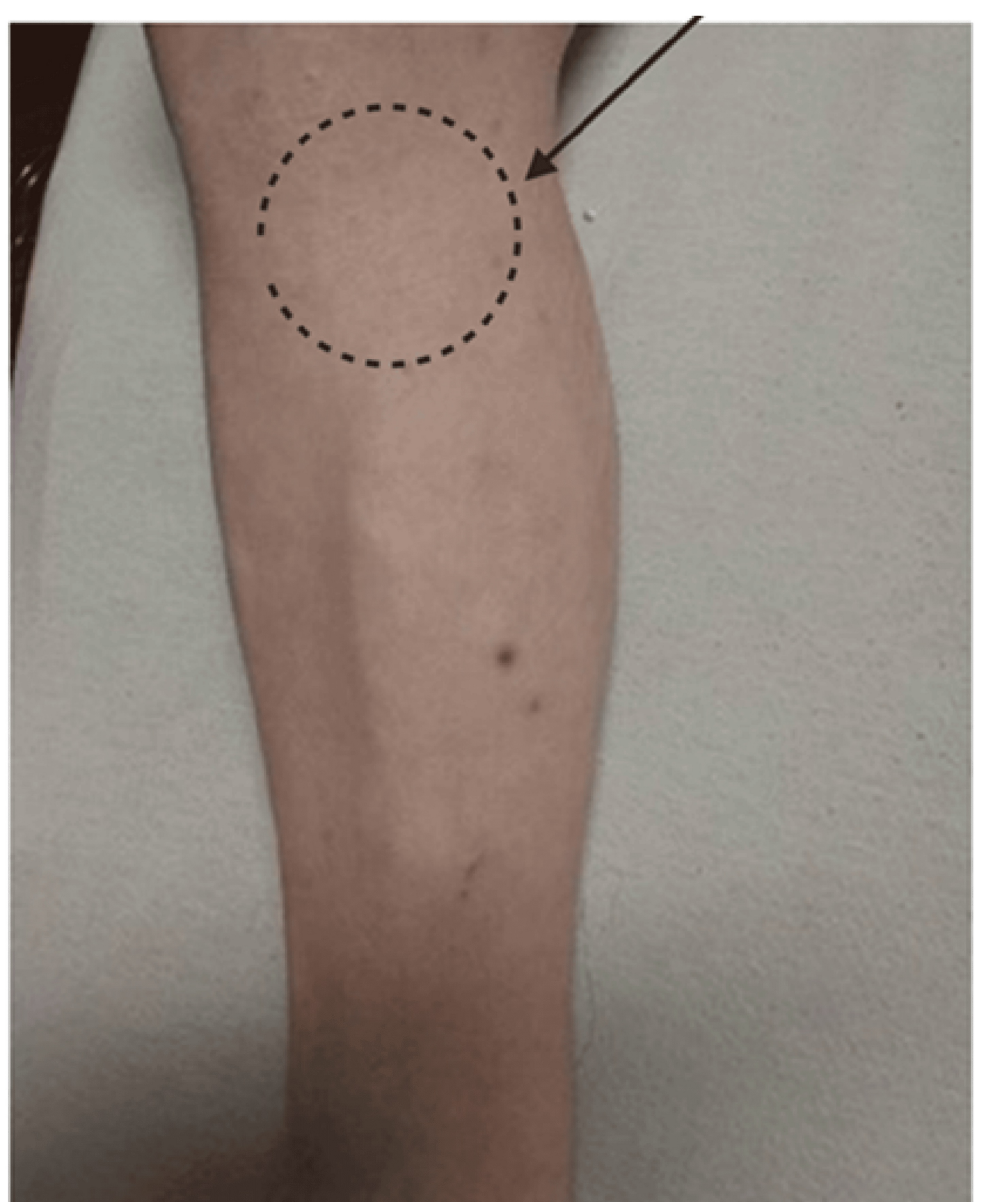
Post-treatment clinical appearance demonstrating significant resolution of inflammatory lesions (arrow and halo) following topical corticosteroid therapy.

**Figure 3:**
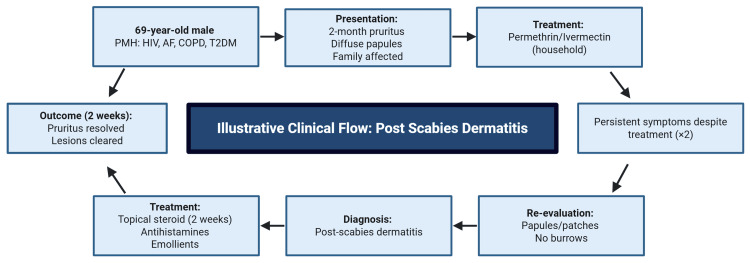
Illustrative clinical flow. This image is created by the author of this study using BioRender software. PMH: past medical history; AF: atrial fibrillation; COPD: chronic obstructive pulmonary disease; T2DM: type 2 diabetes mellitus

The patient was treated with a medium-potency topical corticosteroid (triamcinolone 0.1%) applied twice daily for two weeks, oral antihistamines for symptomatic relief, and regular emollient therapy. The patient was reassured regarding the absence of active infestation and educated about the persistence of pruritus and dermatitis for several weeks due to the inflammatory response. Two weeks later, the patient was followed in the outpatient setting, with resolution of pruritus and inflammatory skin lesions (Figure 2). No further scabicidal therapy was required. The overall clinical course, including symptom onset, treatment interventions, diagnostic reassessment, and resolution, is summarized in Figure 3.

## Discussion

Scabies is primarily an immunologically mediated condition in which symptoms result from host hypersensitivity to mite-derived proteins and fecal material [10]. Continued immune activation and pruritus may persist even after eradication [10,11]. The underlying mechanism involves a delayed-type IV hypersensitivity reaction mediated by T lymphocytes in response to persistent mite-derived antigens within the skin. This immune response leads to continued cytokine release, including interleukin-2 and interferon-γ, resulting in sustained inflammation even after parasite eradication. In some cases, this immune activation may also involve eosinophils and mast cells, contributing to pruritus and eczematous changes [7,12]. Post-scabies dermatitis, therefore, reflects an inflammatory sequela rather than ongoing infestation [7,10]. The persistence of pruritus after treatment completion is common and can last for up to four to six weeks despite adequate therapy [3]. Nodular scabies, another hypersensitivity manifestation, can last longer and is more common in children [13].

The main diagnostic challenge is distinguishing post-scabies dermatitis from reinfestation or inadequate treatment [12]. A structured comparison of clinical and dermoscopic features distinguishing active scabies infestation from post-scabies dermatitis is summarized in Table 1, highlighting key differences such as the presence of burrows, visualization of mites on dermoscopy, and response to prior therapy. The markedly elevated CRP in this case requires careful interpretation. Post-sabies dermatitis is a localized cutaneous hypersensitivity reaction and is not typically expected to cause marked elevation in systemic inflammatory markers. In this patient, the elevated CRP and ESR were attributed to concurrent pneumonia rather than the dermatologic process. Importantly, there was no clinical evidence of secondary bacterial skin infection, such as purulence, warmth, fluctuance, honey-colored crusting, rapidly expanding erythema, or cellulitis. These laboratory findings, therefore, did not support the diagnosis of post-scabies dermatitis but were included to document the broader clinical context and exclusion of alternative cutaneous infectious complications.

**Table 1:** Clinical and dermoscopic features distinguishing active scabies infestation from post-scabies dermatitis. This table summarizes the key clinical and dermoscopic features that help differentiate active scabies infestation from post-scabies dermatitis. Active infestation is characterized by the presence of mites, burrows, and ongoing transmission, whereas post-scabies dermatitis represents a delayed type IV hypersensitivity reaction to retained mite antigens following successful eradication. Dermoscopic findings are particularly useful, as visualization of the characteristic “delta wing jet sign” confirms active infestation, while its absence supports a post-inflammatory process. Accurate differentiation is essential to avoid unnecessary retreatment and to guide appropriate anti-inflammatory management. This table was created by the authors based on the synthesis of the current literature and clinical expertise.

Feature	Active scabies infestation	Post-scabies dermatitis
Presentation	Before or during treatment	After appropriate treatment completion
Pathophysiology	Active infestation by *Sarcoptes scabiei*	Type IV hypersensitivity to retained antigens
Contagious	Common (active transmission)	Absent after treatment of contacts
Clinical findings		
Pruritus	Intense, often nocturnal	Persistent, may be less severe, but ongoing
Lesion type	Erythematous papules, burrows, vesicles, nodules	Eczematous plaques, papules, nodules
Distribution	Typical sites (interdigital spaces, wrists, axillae, genitals)	May overlap, but often more diffuse or nonspecific
Burrows	Present	Absent
New lesions	Continually appearing	No new burrows or classic lesions
Dermoscopic findings		
Dermoscopy - mite (delta wing jet sign)	Present	Absent
Dermoscopy - eggs/scybala	May be present	Absent
Response to scabicides	Improves with treatment	No improvement with additional scabicides
Management	Permethrin/ivermectin	Topical corticosteroids, antihistamines, and emollients
Duration	Persists until treated	Can last 2-6 weeks, sometimes longer, after treatment

Importantly, the clinical presentation of post-scabies dermatitis may closely resemble active infestation, with persistent pruritus and similar distribution of lesions, making differentiation based solely on symptoms particularly challenging [3]. Key distinguishing features of post-scabies dermatitis include absence of new burrows, lack of mite visualization, and the persistence of symptoms despite appropriate treatment adherence and contact management [8]. The absence of mites, eggs, or scybala on dermoscopic evaluation further supports the diagnosis. Diagnosis is therefore primarily clinical, supported by dermoscopic findings and the absence of new burrows. Laboratory evaluation may further assist in excluding alternative diagnoses or confirming resolution of infestation; a summary of relevant laboratory findings and their interpretation in post-scabies dermatitis is provided in Table 2.

**Table 2:** Laboratory evaluation in post-scabies dermatitis. AST: aspartate aminotransferase; ALT: alanine aminotransferase

Test	Result	Reference range	Interpretation
White blood cell count	9.1×10⁹/L	4.0-11.0×10⁹/L	Within normal limits
Absolute eosinophil count	0.09×10⁹/L	0-0.5×10⁹/L	Normal (no eosinophilia)
Hemoglobin	12.2 g/dL	13-17 g/dL	Mild anemia (nonspecific)
Platelet count	406×10⁹/L	150-400×10⁹/L	Mild thrombocytosis (reactive)
C-reactive protein (CRP)	141 mg/L	<5 mg/L	Markedly elevated; attributable to concurrent pneumonia
Erythrocyte sedimentation rate (ESR)	48 mm/h	<20-30 mm/h	Elevated; interpreted in the context of concurrent pneumonia
Sodium	133 mmol/L	135-145 mmol/L	Mild hyponatremia
Creatinine	1.28 mg/dL	0.6-1.3 mg/dL	Normal to mildly elevated
Liver function tests (AST/ALT)	10/11 U/L	Normal	Normal
Serum IgE	Not performed	-	Not routinely required
Skin scraping for mites/ova	Not performed	-	Diagnosis based on clinical course
Bacterial culture	Not performed	-	No clinical evidence of infection

Incorrect permethrin application, untreated close contacts, or emerging resistance remain important considerations in true treatment failure [3,4,12]. A recent systematic review and meta-analysis evaluating scabies treatment outcomes highlighted variability in reported failure rates and underscored the importance of distinguishing persistent symptoms from true therapeutic failure [14,15].

Repeated and unnecessary scabicidal treatment may also increase the risk of medication-related adverse effects, including irritant dermatitis and neurotoxicity with excessive ivermectin use. Additionally, repeated treatments contribute to increased healthcare costs and may exacerbate patient anxiety and psychological distress due to persistent concern about ongoing infestation [4]. Recognizing the hypersensitivity basis of post-scabies dermatitis allows for appropriate management with topical corticosteroids, antihistamines, and emollients rather than additional scabicides [16].

Post-scabies dermatitis should also be distinguished from irritant dermatitis related to scabicidal therapy and from nonspecific eczematous dermatitis. Irritant dermatitis from permethrin or repeated topical therapy is usually related to treatment exposure itself and may present with burning, xerosis, erythema, or worsening dermatitis in areas of application. Nonspecific eczematous dermatitis may overlap clinically but lacks the preceding history of confirmed scabies exposure and treatment response pattern [14].

The patient’s history of HIV also raises the question of whether immune status may have contributed to the persistence or morphology of the post-treatment eruption. Although his HIV was controlled on antiretroviral therapy, altered immune responses in patients with HIV may influence the clinical expression of scabies and the inflammatory response after treatment. In immunocompromised patients, persistent pruritus after scabies treatment should prompt careful evaluation for ongoing infestation, crusted scabies, and secondary infection before diagnosing post-scabies dermatitis [17]. In this case, clinical and dermoscopic findings, together with the response to anti-inflammatory treatment, supported a diagnosis of post-scabies dermatitis rather than persistent infestation.

Some limitations encountered in this case include the absence of skin scraping, so microscopic confirmation of mite eradication was not available. In addition, although dermoscopy is a useful adjunctive tool, it is not perfectly sensitive and may show false-negative results. Therefore, post-scabies dermatitis was not diagnosed based on negative dermoscopy alone, but rather on the overall clinical course, including appropriate scabicidal therapy, treatment and resolution of close contacts, absence of new burrows or classic scabetic lesions, lack of features suggestive of crusted scabies, and complete improvement with anti-inflammatory therapy without additional scabicide use.

Our case highlights the need for thorough follow-up evaluations after treatment. Confirmation of treatment adherence through no new burrows, treatment of close contacts, and negative dermoscopic findings provided diagnostic support for post-scabies dermatitis. Early recognition and patient reassurance are essential to prevent overtreatment and reduce psychological distress related to concerns of persistent contagion.

## Conclusions

This case highlights the practical importance of recognizing post-scabies dermatitis as a cause of persistent pruritus and inflammatory skin lesions after adequate scabicidal therapy. Persistent symptoms alone should not be interpreted as treatment failure or active infestation, particularly when there are no new burrows or classic scabetic lesions and when close contacts have improved after treatment. In this patient, the absence of clinical and dermoscopic findings suggestive of active infestation, the lack of features of crusted scabies or secondary bacterial infection, and improvement with topical corticosteroids and emollients without further scabicidal therapy supported the diagnosis of post-scabies dermatitis. Awareness of this post-treatment inflammatory reaction may help clinicians avoid unnecessary retreatment, treatment-related irritation, and patient anxiety, while still maintaining careful follow-up in patients with immunocompromising conditions or atypical presentations.
